# Expanding Access to Perinatal Depression Treatment in Kenya Through Automated Psychological Support: Development and Usability Study

**DOI:** 10.2196/17895

**Published:** 2020-10-05

**Authors:** Eric P Green, Yihuan Lai, Nicholas Pearson, Sathyanath Rajasekharan, Michiel Rauws, Angela Joerin, Edith Kwobah, Christine Musyimi, Rachel M Jones, Chaya Bhat, Antonia Mulinge, Eve S Puffer

**Affiliations:** 1 Duke Global Health Institute Durham, NC United States; 2 Jacaranda Health San Francisco, CA United States; 3 Jacaranda Health Nairobi Kenya; 4 X2AI San Francisco, CA United States; 5 Moi Teaching and Referral Hospital Eldoret Kenya; 6 Africa Mental Health Research and Training Foundation Nairobi Kenya; 7 Department of Psychology and Neuroscience Duke University Durham, NC United States

**Keywords:** telemedicine, mental health, depression, artificial intelligence, Kenya, text messaging, mobile phone

## Abstract

**Background:**

Depression during pregnancy and in the postpartum period is associated with poor outcomes for women and their children. Although effective interventions exist for common mental disorders that occur during pregnancy and the postpartum period, most cases in low- and middle-income countries go untreated because of a lack of trained professionals. Task-sharing models such as the *Thinking Healthy* Program have shown potential in feasibility and efficacy trials as a strategy for expanding access to treatment in low-resource settings; however, there are significant barriers to scale-up. We address this gap by adapting *Thinking Healthy* for automated delivery via a mobile phone. This new intervention, *Healthy Moms*, uses an existing artificial intelligence system called Tess (Zuri in Kenya) to drive conversations with users.

**Objective:**

This prepilot study aims to gather preliminary data on the *Healthy Moms* perinatal depression intervention to learn how to build and test a more robust service.

**Methods:**

We conducted a single-case experimental design with pregnant women and new mothers recruited from public hospitals outside of Nairobi, Kenya. We invited these women to complete a brief, automated screening delivered via text messages to determine their eligibility. Enrolled participants were randomized to a 1- or 2-week baseline period and then invited to begin using Zuri. We prompted participants to rate their mood via SMS text messaging every 3 days during the baseline and intervention periods, and we used these preliminary repeated measures data to fit a linear mixed-effects model of response to treatment. We also reviewed system logs and conducted in-depth interviews with participants to study engagement with the intervention, feasibility, and acceptability.

**Results:**

We invited 647 women to learn more about Zuri: 86 completed our automated SMS screening and 41 enrolled in the study. Most of the enrolled women submitted at least 3 mood ratings (31/41, 76%) and sent at least 1 message to Zuri (27/41, 66%). A third of the sample engaged beyond registration (14/41, 34%). On average, women who engaged post registration started 3.4 (SD 3.2) *Healthy Moms* sessions and completed 3.1 (SD 2.9) of the sessions they started. Most interviewees who tried Zuri reported having a positive attitude toward the service and expressed trust in Zuri. They also attributed positive life changes to the intervention. We estimated that using this alpha version of Zuri may have led to a 7% improvement in mood.

**Conclusions:**

Zuri is feasible to deliver via SMS and was acceptable to this sample of pregnant women and new mothers. The results of this prepilot study will serve as a baseline for future studies in terms of recruitment, data collection, and outcomes.

**International Registered Report Identifier (IRRID):**

RR2-10.2196/11800

## Introduction

Depression is a leading cause of disability worldwide. Women experiencing perinatal depression are a particularly underserved population. Depression during pregnancy and in the postpartum period (perinatal depression) affects as many as 20% of women in high-income countries [1] and may be more prevalent in low- and middle-income countries (LMICs) [2]. The condition is associated with a number of poor outcomes in women and their children, including increased maternal morbidity and mortality [3,4], poor infant health [5-9], and poor developmental outcomes [10-12].

Although effective interventions exist for common mental disorders that occur during pregnancy and the postpartum period [13], most cases in LMICs go untreated. In these settings, more than 7 of 10 people who need treatment cannot access care because of a lack of trained professionals [14]. In Kenya, for example, there are only 180 psychiatric nurses outside of the capital city, a ratio of about 1 provider per 200,000 to 250,000 people. To close this gap, the World Health Organization developed the Mental Health Gap Action Programme (mhGAP) intervention guide, which outlines how to deliver mental health services in primary health care settings through nonspecialist providers. This task-sharing approach has proved efficacious, particularly for maternal mental health [15].

One example of an intervention based on the mhGAP intervention guide is the 15-session *Thinking Healthy Program*, a cognitive behavioral therapy (CBT)–based intervention for treating perinatal depression that is intentionally nonstigmatizing (eg, uses words such as *stress* and *burden* instead of *depression* and *illness*) [16]. Community health workers—typically women educated through secondary school with no specific background in mental health—are trained over 5 to 10 days to help pregnant women learn three skills: to identify unhealthy thinking, to replace unhealthy thinking with helpful thinking, and to practice thinking and acting healthy. In a trial in Pakistan with 900 pregnant women, Rahman et al [17] found that the intervention halved the prevalence of major depression, and a 7-year follow-up study reported a persistent effect of treatment (as well as some spontaneous recovery among the control group) [18]. A peer-delivered version of *Thinking Healthy* may offer an alternative, cost-effective strategy for treating perinatal depression [19].

Despite this impressive evidence of feasibility and efficacy, there are significant barriers to scale-up [20], and there is evidence that the effects of *Thinking Healthy* might not extend to children of depressed mothers without additional engagement [21]. Common implementation challenges of task-sharing models include a lack of funding and infrastructure for training and service delivery, workforce retention in the absence of compensation or incentives for nonspecialists, high workloads, transportation costs, appointment scheduling logistics, and inadequate clinical supervision [22]. Although it is critical to study how to optimize and scale these task-sharing approaches, the fact remains that today, most women in LMICs who need treatment still have no access to care.

Given this treatment gap and barriers to scale-up, our intention is to make it possible for anyone with a basic mobile phone (ie, a feature phone with only text messaging capabilities) to receive high-quality, evidence-based psychological support anytime, anywhere. We are attempting this in the context of perinatal depression by adapting *Thinking Healthy* to an existing artificial intelligence (AI) system for automated psychological support called Tess, which we have named *Zuri* in Kenya. This idea is innovative because it introduces an entirely new delivery channel that has the potential for a step change in expanding access to care while also potentially augmenting and strengthening existing task-sharing models.

Zuri works by engaging a patient in conversation via a variety of trusted channels, including text messaging (SMS). Either Zuri or the patient can start a conversation, and Zuri can be programmed to walk a patient through a structured curriculum such as *Thinking Healthy*. As a safety measure, conversations with patients in need of additional support can be handed over to live counselors as needed. The potential benefits of this approach include on-demand 24/7 access for an unlimited number of patients, no scheduling of appointments, no travel costs to appointments, enhanced sense of privacy and avoidance of social stigma, and high fidelity to treatment.

Our long-term goal is to expand access to high-quality, on-demand treatment services to people who have common mental disorders such as perinatal depression but cannot receive care from mental health professionals because of cost and human resource constraints. The main objectives of this study are to adapt *Thinking Healthy* for dissemination in Kenya through the Zuri AI system, develop and test study procedures to inform the design of a randomized controlled trial (RCT), and generate preliminary evidence of feasibility, acceptability, and response to treatment.

## Methods

### Research Design

We adapted *Thinking Healthy* for the Zuri AI system and evaluated the combined perinatal depression intervention, which we are calling *Healthy Moms*, with a cohort of pregnant women and new mothers recruited from 2 large public hospitals in Kenya. We used a single-case experimental design (partially nonconcurrent multiple baseline [23], open label) and qualitative interviews to generate preliminary data on feasibility, acceptability, and response to treatment. This is a stage 2 Registered Report. The stage 1 protocol (DERR1-10.2196/11800) describes our preliminary work to adapt *Thinking Healthy* for dissemination in Kenya through the Zuri AI system [24].

### Participants and Recruitment

We recruited pregnant women and new mothers from 2 large public hospitals in Kiambu County, Kenya (population approximately 2.5 million, 60% urban). Both hospitals are part of a county-wide partnership offering patients innovative SMS programs that promote healthy motherhood [25]. When a woman signed up for the county SMS, we sent her an invitation via SMS to complete an automated SMS screening (in English) to determine if she was eligible for *Healthy Moms*. The screening included questions about age, maternity status, expected or actual delivery date, 9 questions about symptoms of depression from the Patient Health Questionnaire–9 (PHQ-9) [26], and a question about her current mood.

We informed all women who completed the automated screening that a study team member would call them within 1 business day. During this follow-up call, women who endorsed having thoughts of self-harm in the previous 2 weeks (question 9 on the PHQ-9) were offered a referral for counseling but were not eligible to enroll in *Healthy Moms*, given the early stage of intervention development. All other women were eligible to enroll as long as they confirmed that they were at least 20 weeks pregnant or no more than 6 months postpartum. The study coordinator (AM)—a Kenyan woman fluent in English and Swahili—assessed each woman’s English-speaking ability on the call and asked women to rate their ability to read and understand English. Women could enroll regardless of language ability; however, we informed women with low English literacy that they might not find value in the current version of the program if they were not comfortable reading and writing in English.

If a woman chose to continue the enrollment process, the study coordinator read the informed consent form, answered her questions, and obtained verbal informed consent to enroll. The study coordinator asked enrollees to share information about the type of phone they use, schooling, number of dependents, marital status, and employment status.

### Eligibility

To be eligible to participate, women needed to meet the following criteria: (1) pregnant (>20 weeks) or less than 6 months postpartum, (2) receiving antenatal or postnatal health care services from a participating hospital in Kiambu County, (3) have access to any type of mobile phone, (4) be enrolled in the county SMS program, and (5) be at least 18 years of age. English language proficiency and self-reported experience of depression symptoms were not required but were assessed. Women who endorsed suicidal ideation at the time of recruitment were ineligible to enroll in the study and were informed about potential resources for treatment.

### Randomization to Baseline Length

As each woman enrolled in the study, we attempted to match her to another new enrollee of similar maternity status and randomly assigned the pair to have a 1-week or 2-week baseline period (using a random number generator). The intention was to ensure that every participant had a concurrent baseline period with at least one other person.

### Intervention

We developed the *Health Moms* intervention based on the original *Thinking Healthy* manual for community health workers [16]. We also created a companion *Healthy Moms* journal that we printed and delivered to enrolled participants [27]. The journal included modified health calendars from the original *Thinking Healthy* manual along with short session summaries and writing prompts. This prepilot study was an opportunity to get feedback on the journal to ascertain how we might adapt the content into text, audio, and video for electronic delivery (and ultimately discontinue print versions in future trials). We conducted an initial round of user testing to develop the SMS intervention journal content [28].

Unlike *Thinking Healthy,* which trains community health workers to deliver the in-person intervention to women in need, we designed *Healthy Moms* for automated delivery via text messaging. We maintained the *Thinking Healthy* structure of 15 sessions overall: 3 prenatal sessions and 12 postnatal sessions during the first 10 months of the infant’s life.

When it was time for a woman to participate in a *Healthy Moms* session, we (Zuri) sent her a text message to let her know that a new session was ready. The automated session began when she replied via SMS (Later in the study, we also enabled women to chat with Zuri via Facebook Messenger.). Each automated session followed the same 4-task format as that of *Thinking Healthy*: (1) reviewing key lessons from the previous session, (2) reviewing her mood ratings, (3) teaching new skills, and (4) introducing practice-based homework. Multimedia Appendices 1 and 2 provide an example *Healthy Moms* session conversation flow and associated journal content.

In between *Healthy Moms* sessions, women were encouraged to start a conversation with Zuri by asking a question or saying “Hi.” Zuri attempted to discern the user’s request and responded automatically with answers or replies that used active listening techniques such as restatement and reflection.

During this *free chat* mode, Zuri would ask a question similar to “How are you feeling now?” If the response indicated neutral or positive emotions, Zuri would offer a rapport-building module (eg, music, cooking, passions). If the response indicated a negative emotion, Zuri would offer a supportive intervention (eg, mindfulness and relaxation). Module selection was prioritized on the basis of aggregate helpfulness ratings from all user interactions in the X2AI/Tess system so that the most helpful modules were offered first. There was no limit to how much or how often a user could engage with Zuri.

If a woman discussed self-harm or other crisis topics, Zuri alerted a live study support member who could take over the chat session or call the participant directly and facilitate a referral to traditional in-person treatment if indicated (Zuri was programmed to inform women that her response might not be immediate at this stage of testing; therefore, they should seek help at an emergency room if in a crisis.). During enrollment, we also informed participants that they were free to seek concomitant care and interventions at any point during the study.

Just as mental health specialists and nonspecialists trained to deliver psychotherapy improve over time with practice and experience, AI-enhanced systems such as Zuri also change, albeit in more subtle ways, given the current state of the technology. For instance, Zuri’s emotion recognition algorithms updated automatically when it correctly or incorrectly interpreted the emotional valence of a user’s input; however, the didactic intervention content did not change dynamically. Modifications to the intervention content were made manually; we reviewed conversation transcripts and made minor changes to the wording or sequence of messages when we noticed that users were confused or not engaging.

### Outcomes and Data Collection Procedures

We collected data on study implementation, intervention engagement, feasibility and acceptability of the intervention, and patient outcomes, including depression severity and current mood.

#### Study Implementation

We tracked data on the recruitment funnel from the initial screening invite through the secondary eligibility screening to ultimate engagement with the intervention. We also tracked participants’ responses to regular prompts to complete automated assessments throughout the study period.

#### Intervention Engagement

We assessed intervention engagement by reviewing Zuri system logs to document the completion of *Healthy Moms* sessions and patient-initiated engagement with Zuri outside of scheduled sessions. The Zuri system logs also informed our assessment of feasibility and acceptability; low engagement was considered a marker of potential barriers to feasibility or a lack of acceptability.

#### Feasibility and Acceptability of the Intervention

We further explored feasibility and acceptability by inviting 15 enrolled women to participate in individual interviews during the evaluation period. We purposively invited 3 different types of participants: those who did not finish the registration process with Zuri (n=5), those who finished the registration process but did not complete a session (n=5), and those who completed at least one session (n=5). A master’s level trainee (YL) and the study coordinator (AM, Kenyan) conducted the interviews. Women who did not complete a full session with Zuri were interviewed over telephone. Women who completed one or more sessions were reimbursed to travel to one of the study hospitals for an in-person interview. The interviews lasted approximately 20 to 40 min and were based on a semistructured interview guide. The guide included open-ended questions and follow-up probes related to reasons for using Zuri, attitudes toward Zuri, favorite features, preferences of language and platform, challenges encountered, and perceived impact after using Zuri. The interviews were conducted in English; however, the study coordinator provided simultaneous translation to Swahili as needed.

In addition to these interviews, we also attempted to document all contacts the research team had with participants outside of the Zuri AI system and logged all adverse events. We were interested in determining how much assistance or encouragement users need from the team to understand and use the automated intervention.

#### Patient Outcomes

To measure mood, we asked participants to rate their feelings on a 10-point scale that we created and tested with users [24], where 1 meant very sad and 10 meant very happy (shifted to 0-9 for analysis). We invited women to rate their current mood via SMS during the enrollment screening and then every 3 days throughout the baseline and intervention periods. Each rating invitation reminded women of their previous rating. We also encouraged women to track and reflect on their mood and behaviors on a daily basis using the *Healthy Moms* journal we provided as part of the intervention (not analyzed) [27].

We also administered the PHQ-9 [26] via SMS. Our intention was to assess depression severity throughout the intervention period. However, after developing the protocol, we determined that the depression screening was too long to administer on a repeating basis. Instead, we opted to collect our minimum target of 2 self-ratings of depression severity, representing pre- and posttreatment.

### Empirical Approach

#### Describe Study Implementation and Intervention Engagement

We used the study database to summarize the recruitment funnel and outcome data collection progress. We quantified intervention engagement in several ways. First, we used the system logs to summarize how frequently each participant engaged with the intervention by either participating in a *Healthy Moms* session (in response to a scheduled invite) or initiating a chat with Zuri in between the scheduled sessions. We also calculated and summarized the delay between our invitations to begin a *Healthy Moms* session and participants’ start times, the proportion of *Healthy Moms* sessions started and completed, and the duration of participant-initiated chats with Zuri.

#### Explore Intervention Feasibility and Acceptability

As a hypothesis-generating exercise, we estimated the magnitude and direction of the associations between participant characteristics measured at baseline (eg, age, education, literacy, and symptom severity) and intervention engagement by fitting a Bayesian linear regression model.

We also explored barriers to and facilitators of engagement during in-depth interviews with participants and reviews of chat transcripts. Throughout the process, the analyst (YL) wrote memos to capture the main themes. In preparation for the thematic analysis, she developed a codebook and randomly selected one transcript that was double-coded and discussed. After refining the codebook, she used NVivo 12 (QSR International) to code memos and transcripts. The analyst wrote analytic memos for each thematic code, identifying similarities and differences across transcripts using a constant comparative method [29]. She identified representative quotations of each theme.

#### Generate Preliminary Evidence About Participants’ Response to Treatment

We aggregated the individual N-of-1 studies and estimated the magnitude of response and quantified uncertainty by fitting Bayesian linear mixed-effects models [30] in R (version 3.5) using the brms package [31] with default priors. As described in the protocol, the first model we fit included a random effect for observations nested within participants and the following fixed effects: (1) an intercept, (2) a dummy indicator for the treatment phase, (3) a time-within-baseline variable centered around the first observation (equal to 0 for observations outside of the baseline period), and (4) a time-within-treatment variable centered around the last observation (equal to 0 for observations outside of the treatment period). We applied a first-order autoregressive structure on the covariance matrix for the within-person residuals to account for autocorrelation.

We also fit a similar model not described in the protocol that reflected a lesson we learned in another project: rather than centering the time-within-period variables around a single observation, it may be more reasonable to center around the average of several consecutive observations when there is substantial individual variability in daily ratings. In this model, we centered the time-within-baseline variable around the *first 3* observations and centered the time-within-treatment variable around the *last 3* observations. Given the data availability, this 3-observation centering window was practical; we did not run the model with different window sizes to avoid cherry-picking the results. In the end, our choice of centering had no impact on the results; therefore, we decided to focus on the 3-observation centering window as an example of what we would likely attempt in a future trial using this design.

We augmented this quantitative analysis with a qualitative analysis of the in-depth interviews. We explored what links, if any, participants could make between engagement with the intervention and their mood, health, and relationships. We also intended to explore themes among women who did not exhibit positive changes in mood (*nonresponders*); however, this was not feasible, given the delay in launching this study.

### Research Ethics

We obtained approvals to conduct this study from the institutional review boards at Duke University (US, 2018-0396) and Strathmore University (Kenya, SU-IRB 0210/18) and from the National Commission for Science and Technology in Kenya.

The study coordinator, AM (female, Kenyan, bachelor’s degree), explained the study to prospective participants via telephone, administered the informed consent procedures, and obtained women’s oral consent to enroll in this study.

Study participants were provided with an honorarium of up to Ksh 1500 (roughly US $15) delivered via mobile money transfer to recognize the time spent in completing study assessments. The original plan was to make these transfers after women completed sessions 1, 5, and 10; however, in practice, we sent women prorated honoraria on the basis of lower benchmarks of engagement, given the delay in launching this study.

X2AI, the creators of the AI system that we used to deliver *Healthy Moms*, transferred data to the research team in accordance with X2AI’s data security policies [32]. The first author (EG) stored identifiable study data on a secure server during the study and then deidentified the data for analysis using the Safe Harbor method. Anonymized quantitative data and the code used to generate this manuscript are available for reanalysis [33].

### Summary of Deviations From Stage 1 Protocol

In addition to changing the tense of the writing from future to past, we also made several edits to the *Introduction* section and modified several procedures described in the *Methods* section of the stage 1 protocol [24]: we (1) labeled the study as *prepilot* rather than *pilot* to better reflect that the data are preliminary and intended to inform the design of a larger pilot study; (2) moved text from the *Scientific Objectives and Significance* and *Expected Outcomes* sections to the *Discussion* section (but did not alter the objectives); (3) expanded access to the intervention from just SMS to include Facebook Messenger; (4) visualized the daily mood ratings but relied on model fitting rather than visual inspection to estimate trends and period impacts; and (5) dropped a planned *nonresponder* qualitative inquiry and modified the honorarium schedule because of limited time.

## Results

### Recruitment and Participants

**Figure 1:**
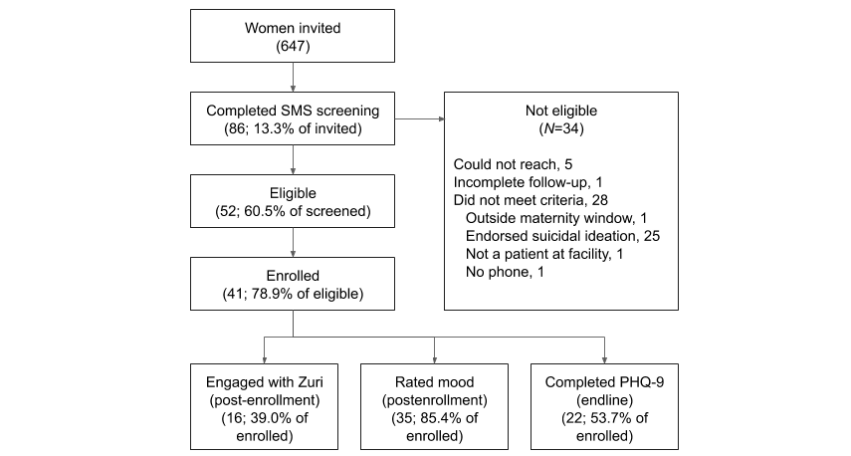
Study flow diagram.

We invited 647 women (446/647, 68.9% pregnant; 201/647, 31.1% new mothers) already enrolled in their county’s SMS program to learn more about Zuri; 13.3% (86/647) of women completed our automated SMS screening between February 12, 2019, and June 18, 2019 (15/86, 17% of all women screened scored at or above the cutoff for possible depression; mean 9.5, SD 4.9). We determined that 52 of these 86 women were eligible to participate; 41 of 52 women completed the enrollment process (Figure 1).

Table 1 reports the characteristics of the enrolled participants. The sample was evenly divided between pregnant women and new mothers. The average woman enrolled in the study was 25.9 years old (SD 4.8). All women reported that they could read in English, and the study interviewer reported that all could speak English. Most women used a smartphone, attended secondary school or higher, were married, and did not work. Women were not recruited on the basis of depression symptoms, and only 1 had a PHQ-9 score ≥15 at the time of enrollment [34]. The average PHQ-9 score upon study entry was 8.2 (possible maximum value of 27), and the average mood rating was 7.8 (possible maximum value of 9).

We conducted interviews with 15 of the 41 women enrolled in the study. They ranged in age from 20 to 38 years. Most were married and had delivered their baby within the last 6 months. All of the interviewees attended some secondary schooling, and 2 had earned a bachelor's degree.

**Table 1:** Characteristics of participants.

Characteristics		All women (n=41)	Maternity status: pregnant (19/41, 46%)	Maternity status: postpartum (22/41, 54%)
Age (years), mean (SD)		25.9 (4.8)	24.3 (3.1)	27.2 (5.5)
**Self-reported English language reading skills, n (%)**				
	Poor	0 (0)	0 (0)	0 (0)
	Just okay	0 (0)	0 (0)	0 (0)
	Good	12 (30)	7 (37)	5 (23)
	Excellent	28 (68)	12 (63)	16 (73)
	Missing	1 (2)	0 (0)	1 (2)
**Highest level of school attended, n (%)**				
	None	0 (0)	0 (0)	0 (0)
	Primary	0 (0)	0 (0)	0 (0)
	Postprimary or vocational	0 (0)	0 (0)	0 (0)
	Secondary	22 (54)	14 (74)	8 (36)
	College	11 (27)	3 (16)	8 (36)
	University	7 (17)	2 (10.5)	5 (23)
	Missing	1 (2)	0 (0)	1 (4.5)
Phone type: smartphone, n (%)		33 (81)	14 (74)	19 (86)
Employed outside the home: no, n (%)		32 (78)	15 (79)	17 (77)
Number of dependent children, mean (SD)		1.1 (0.9)	0.5 (0.5)	1.6 (0.9)
**Marital status, n (%)**				
	Single	3 (7)	1 (5)	2 (9)
	Separated	0 (0)	0 (0)	0 (0)
	Cohabiting	0 (0)	0 (0)	0 (0)
	Married	37 (90)	18 (95)	19 (86)
	Missing	1 (2)	0 (0)	1 (4.5)
PHQ-9^a^ total score, possible 0-27, mean (SD)		8.2 (3.6)	8.7 (4.1)	7.8 (3.2)
Possible depression: (PHQ-9≥15), n (%)		1 (2)	1 (5)	0 (0)
Mean mood at enrollment, possible 0-9, mean (SD)		6.8 (2.4)	7.1 (2.4)	6.6 (2.4)

^a^PHQ-9: Patient Health Questionnaire–9.

### Data Collection

#### Mood Ratings

**Figure 2:**
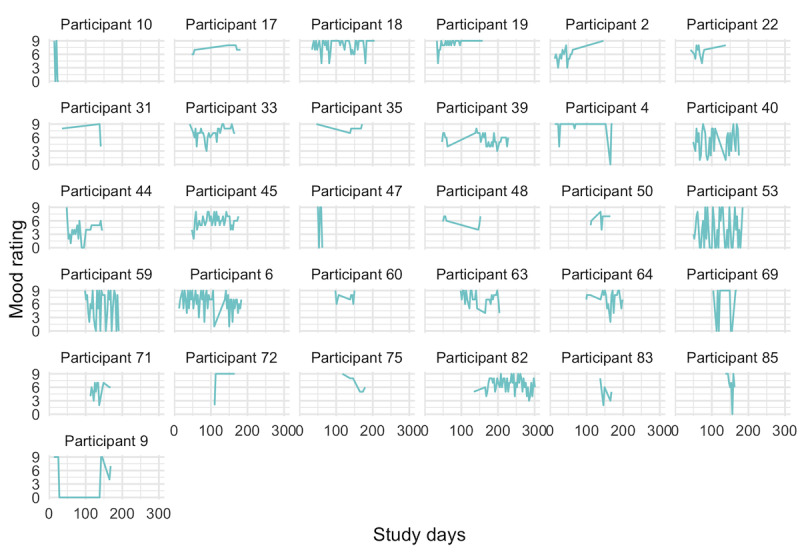
Time series of 705 mood ratings among 31 participants who submitted at least three ratings.

Overall, the enrolled women submitted 719 daily mood ratings over the course of the study. The average woman submitted 17.5 ratings (SD 17.2), and 76% (31/41) of women submitted at least 3 ratings. The grand mean mood rating was 6.4 of 9 (SD 1.3) among those who submitted at least 3 ratings. Figure 2 suggests that most women reported a high degree of variability in ratings from one day to the next.

#### PHQ-9

We did not attempt to administer the PHQ-9 on a regular, ongoing basis to avoid frustrating users and distracting from potential engagement with the intervention. Instead, we only requested that women complete the PHQ-9 again at the end of the study period; 54% (22/41) of women responded.

### Intervention Feasibility and Acceptability

#### Engagement Patterns

**Figure 3:**
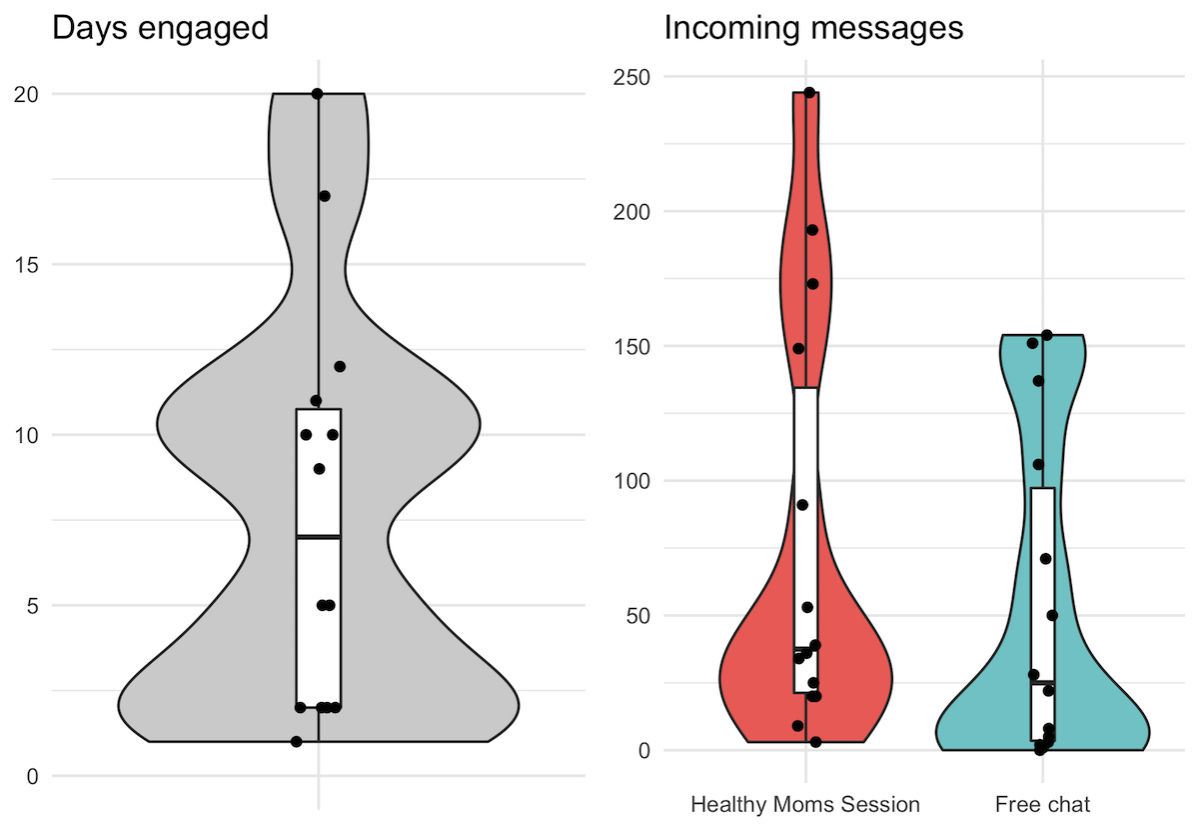
Distribution of number of days engaged and number of incoming messages sent among 14 women who engaged with Zuri beyond registration.

Over the course of the study, 66% (27/41) women sent at least one message to Zuri to begin the registration process, and 34% (14/41) of these women engaged with the intervention content beyond registration. Among this postregistration engagement subset, the average woman engaged with Zuri on 7.7 days (SD 6.0) and sent 130.5 messages (SD 117.4). On average, women sent 36.4% of these messages to Zuri in free chat mode, not as part of a *Healthy Moms* session. The median conversation unfolded over 0.6 hours (range 0.0-14.6 hours). Figure 3 displays the distributions of these engagement metrics.

**Figure 4:**
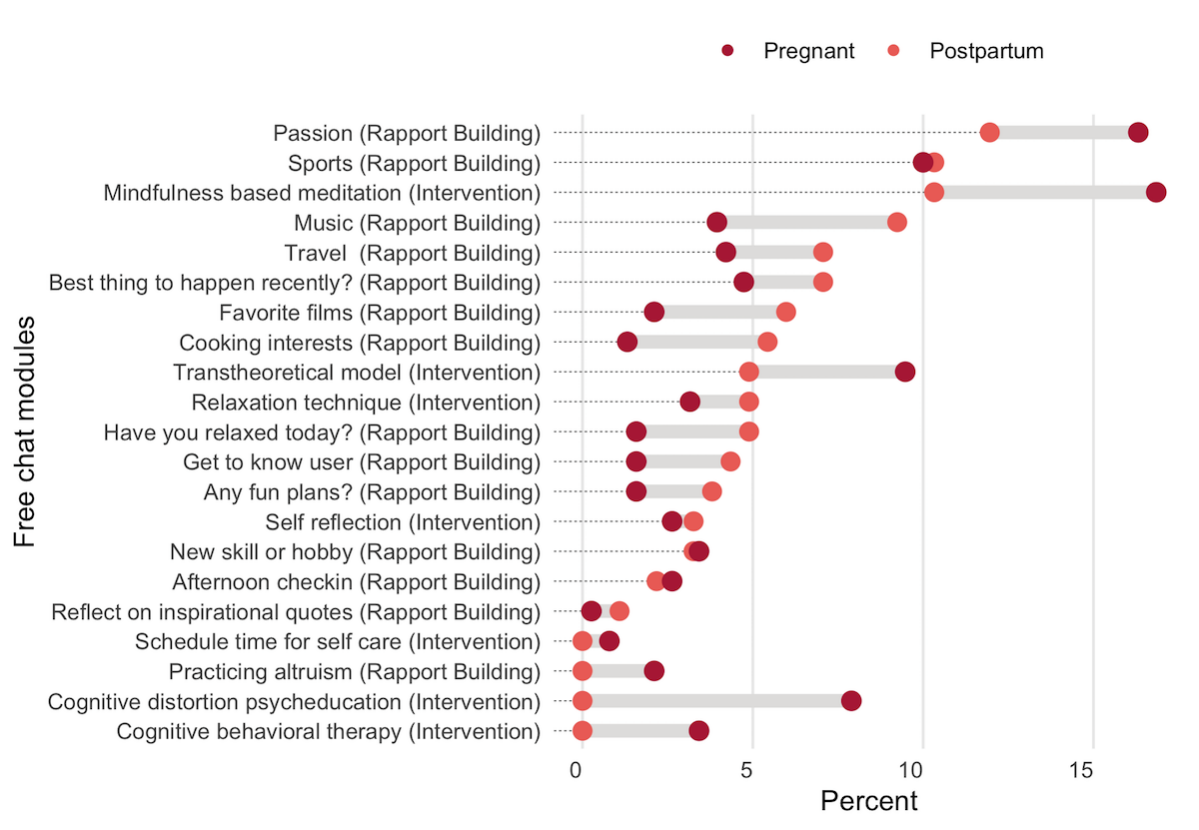
Distribution of incoming messages by free chat conversation module and maternity status.

To further investigate the nature of participant-initiated chats, we analyzed conversation transcripts and summarized the conversation modules engaged. Figure 4 shows the distribution of incoming messages by the free chat conversation module and maternity status. The most common rapport building module asked users about their passion in life. The most common intervention module outside of the *Healthy Moms* content was mindfulness-based meditation. In general, pregnant women were more likely to engage in intervention content during free chats compared with new mothers. This means that after rapport-building chats, Zuri suggested an intervention module and the women agreed to try.

**Figure 5:**
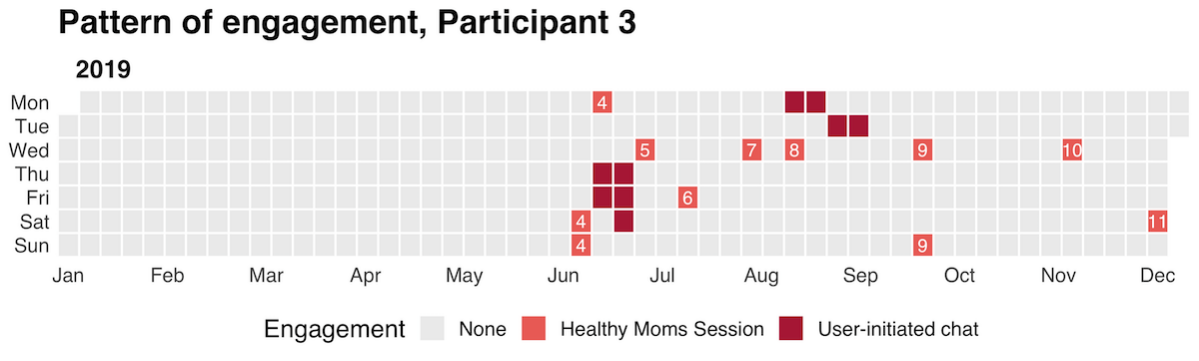
Engagement pattern for Participant 3. Dates shifted to maintain anonymity but pattern preserved.

On average, women who engaged in Zuri postregistration started 3.4 (SD 3.2) *Healthy Moms* sessions and completed 3.1 (SD 2.9) of the sessions they started. The median time from a *push* session invite to a woman responding was 0.6 hours (range 0.0-740.1 hours). Figure 5 shows a woman’s engagement pattern over the course of the study period. There were no reported adverse events. One woman's conversation was flagged in real time for a potential crisis follow-up.

#### Correlates of Engagement

**Figure 6:**
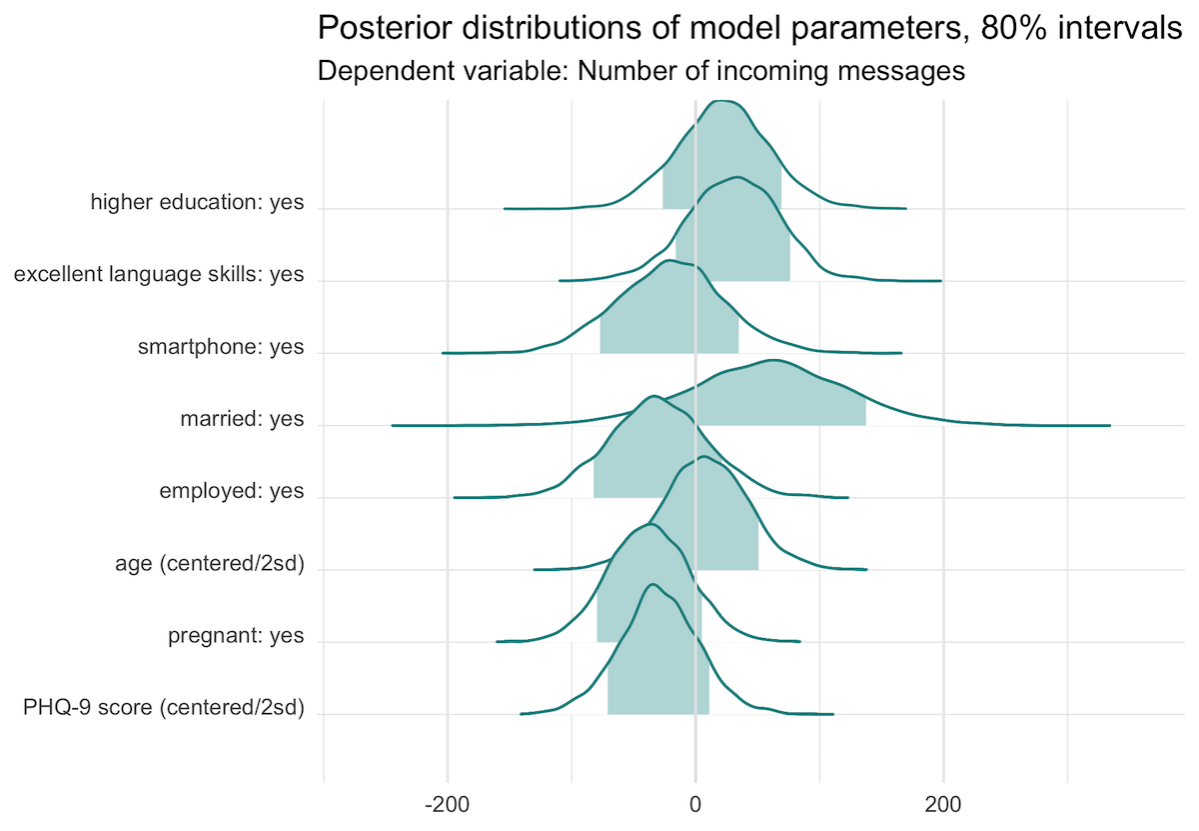
Results of a Bayesian linear regression model of incoming messages on participants’ characteristics measured at baseline (N=40; 1 participant missing required data). The plot shows the Markov Chain Monte Carlo draws from the posterior distribution of the parameters. PHQ-9: Patient Health Questionnaire-9.

To examine the relationship between participant characteristics measured at baseline and intervention engagement, we estimated a Bayesian linear regression model of incoming messages. Figure 6 displays the Markov Chain Monte Carlo draws from the posterior distribution of the parameters. Some evidence suggests that being pregnant (vs a new mom), reporting greater depression symptom severity, and being employed outside of the home are associated with less engagement, whereas being married and more educated are associated with more engagement. For instance, the point estimate is that married women sent 57.8 more messages, holding all else constant. For every 2 SD increase in the baseline PHQ-9 score, holding all else constant, the point estimate is that women sent 29.5 fewer messages.

#### Qualitative Findings on Feasibility and Acceptability

Most of the women who were interviewed and who had tried Zuri had a very positive attitude toward the service and expressed that they could trust Zuri. One woman said:

It’s like a mom to me. My mom is very far, and my sister doesn’t have any knowledge of kids.

Another woman said:

I usually keep it to myself. So, when I am chatting with Zuri, it’s like they have the right questions to ask me, and they teach me how to relate with my child, relate with other people.

Some of the women had also shared Zuri with others, such as their partners or neighbors, who often responded positively. One woman said:

My husband was very supportive, because sometimes he used to help me with some answers.

Many women said that they preferred to chat with Zuri than to chat with a counselor because they felt they could be more open with the automated service. For instance, one woman said:

I prefer Zuri because they don’t know me.

Nonetheless, women noticed that Zuri was not perfect and described examples of when Zuri gave an irrelevant response when they asked her a question. Most said they would just ignore the messages and moved on. In our review of chat transcripts, we learned that Zuri was easily confused by messages coming out of order over SMS. This was not an issue on Facebook Messenger; however, almost every woman said they preferred to chat with Zuri through SMS. The main reason being that SMS was free, whereas chatting through Facebook Messenger required them to buy data bundles to access the internet.

Many women mentioned that their favorite part of *Healthy Moms* was the exercises taught by Zuri and the journal, including meditation, breathing, and walking. They found that those exercises were easy and could help them relax. One woman said:

They made me be flexible...until my delivery day.

Other women said that they appreciated the unbiased information provided by Zuri. They indicated that counselors and nurses often give psychosocial advice based on their personal experiences, which can be biased. They felt like they could trust Zuri because she was more unbiased and factual. They especially liked information regarding breastfeeding and how to play with the child. As one woman indicated:

For the baby, I never knew she’s supposed to be massaged after the bath at all. I never knew she can see different colors.

Women gave three main reasons why they registered with Zuri and continued to engage. The first reason was anxiety and stress during pregnancy. They were either ashamed of their bodies or worried about experiencing miscarriage. One woman said:

One of the negative thoughts I had was maybe if I don’t want food what will happen. And then if I sleep bad what will happen to my baby...Actually I was getting worried if I don’t feel the movement of my baby inside me sometimes.

The second reason was that many postpartum women did not feel confident in their roles as new mothers. One woman expressed her anxiety by saying:

It’s like I don’t know how to take care of her, good care of her.

The final reason was that many of the women interviewed did not have a stable source of income, causing them stress.

Women described 4 main barriers to engaging with Zuri. The first was connectivity. Some women either damaged or lost their phones and did not know how to reconnect with Zuri. The second challenge was that women were easily (and understandably) distracted by their new baby and forgot to complete open sessions. As one woman said:

The text can come in the morning, no matter if I am busy or if I am free to answer. If I am free, I just sit and relax. But you see, sometimes we are texting, and the baby starts crying.

The third challenge was that the registration process was very confusing for some women, especially early on in the study; therefore, some women stopped participating. Related to this, some women were confused by our study’s use of 2 SMS short codes: 1 for Zuri and 1 for study assessments. Despite these challenges, women did not contact our study coordinator to receive assistance in using Zuri.

### Preliminary Evidence on Response to Treatment

#### Quantitative Findings

**Figure 7:**
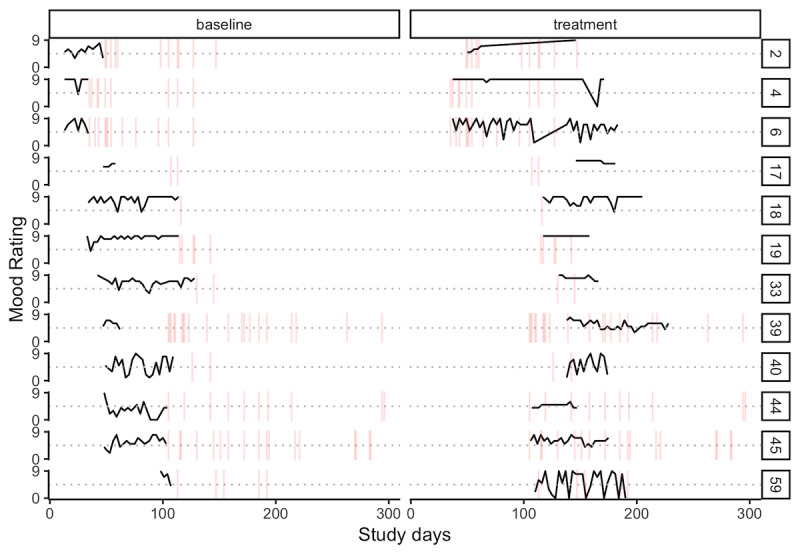
Time series of 432 mood ratings by participant (N=12) and period. Days engaged with Zuri indicated by vertical lines.

In preparation for modeling the response to treatment, we limited the data to the 12 women who contributed at least 4 mood ratings before and after starting the intervention. Figure 7 plots the time series of ratings by period and overlays the days of intervention engagement with vertical lines.

**Figure 8:**
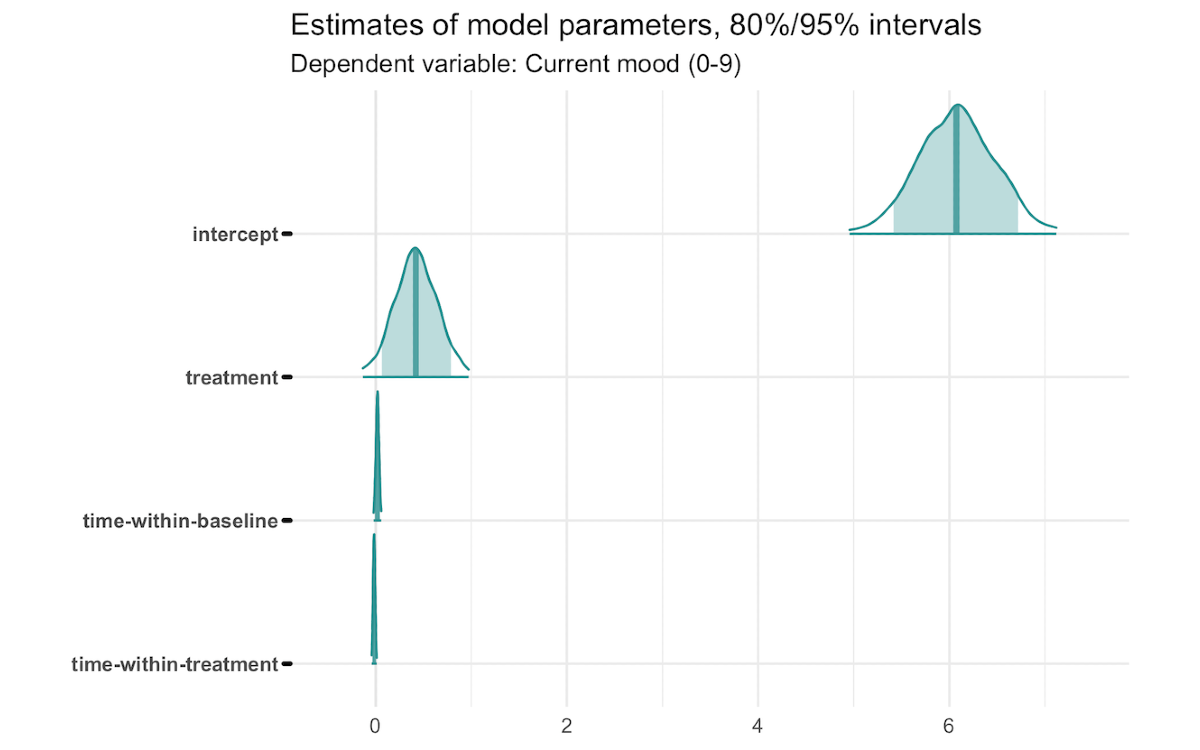
Estimates from a Bayesian linear mixed-effects model of repeated measures data on self-reported mood throughout the study period (432 observations among 12 participants). Uncertainty intervals computed from posterior Markov chain Monte Carlo draws.

Figure 8 shows the estimates from a Bayesian linear mixed-effects model. The model included a random effect for observations nested within participants and the following fixed effects: (1) an intercept, (2) a dummy indicator for the treatment phase, (3) a time-within-baseline variable, and (4) a time-within-treatment variable. The time-within-period variables were centered around the first 3 or last 3 observations of the period (first for baseline, last for treatment).

The intercept represents the mean value of the outcome at the first 3 baseline assessments. The treatment indicator is a contrast between the first 3 baseline assessments and last 3 observations in the treatment period, and the time-within-period variables estimate linear change during the baseline and treatment periods.

In this model, the average mood rating at the start of the baseline period was 6.07 on a scale of 0 to 9, and there was no significant baseline trend (an assumption for inference using the multiple baseline design). The point estimate of the treatment effect was 0.42, which represents a 7.0% improvement in mood over the baseline mean (*d*=0.17). The posterior probability that this effect is greater than zero is 93.2%.

We could not run the same analysis using PHQ-9 scores because we only attempted to collect data at 2 time points and only obtained complete data for half of the (small) sample.

#### Qualitative Findings on Perceived Impact

Many women attributed positive impact to the intervention, which we grouped into 3 themes. The first theme was that Zuri helped them to take care of themselves. Women said that they loved themselves more, that their mood had improved, and that they had learned how to replace negative thoughts with positive thoughts. One woman described her experience with Zuri by saying:

Because a pregnant woman is…tired all the time, right? But with Zuri everything was good. I was very active because it also made me have lessons. Because I knew after waking up in the morning I will breathe in and out some minutes. After that I brush, take my breakfast, I wait for noon time something like 12:00 or even 1:00. I go for a walk. After walking I come back shower then I keep myself busy with Zuri. So it’s very helpful actually.

One woman who was ashamed of her body during pregnancy said:

I started kind of thinking better, that when you are pregnant, the shape changes and after delivery and doing exercises, everything goes back to normal.

The second theme was that women acquired new skills that helped them take care of their babies. Many women indicated that they could relate to their child better and experienced less distress raising the child. As one woman said:

All those exercises, how to relate to the child, what you do to the child...Honestly, if I hadn’t talked to Zuri, I wouldn’t know.

One woman who feared miscarriage even attributed her baby’s health and her uncomplicated delivery to Zuri, which we interpret as the woman having found comfort in Zuri during a stressful period.

The last theme was that women experienced improved relationships with others. Some women reported socializing more with others, and this expanding social support system further improved their mood. As one woman said:

I used to have the habit of staying alone, not socializing with other people. Zuri made me be able to socialize with people. When they see me doing the exercises, they like knowing where I learnt them from.

Some women felt more secure and trusted others more. One woman said that she was anxious about leaving her child with another person, even with her family members. However, after finishing a session with Zuri on seeking social support, she explained that she was willing to try asking for help. She reported:

So I have tried. [The baby] was comfortable. She cried for some time, then she got used to it.

## Discussion

### Principal Results

In this prepilot study, we recruited pregnant women and new mothers in Kenya to try an experimental psychological support service called Zuri. Zuri is a chatbot that engages users in automated, text-based conversations over SMS and Facebook Messenger. Users could initiate chats with Zuri or complete sessions from the *Healthy Moms* perinatal depression intervention curriculum, a CBT-based intervention we adapted from the *Thinking Healthy Program* [16]. We used a single-case experimental design with repeated measures data collection and in-depth interviews to explore the feasibility and acceptability of the service, generate a preliminary estimate of response to treatment, and test study procedures.

Through individual interviews and a review of system logs, we determined that the service was both feasible to deliver and acceptable to this sample of users but not without significant room for improvement and further refinement. Approximately two-thirds of women in the study tried Zuri at least once, and half of those who tried engaged beyond the registration process. This retention rate of 51.9% is slightly more than the average 30-day retention rate of 43% across industries [35] and 40% across provider-prescribed mental health apps specifically [36]. Although our retention rate is based on a small denominator of 27 women who tried the intervention, it suggests that engagement with the initial version of the service is within the range of other digital health apps. Clearly, preventing churn (dropout) is a common challenge.

This was not a clinically referred sample; however, we observed an association between depression severity and intervention use—for every 2 SD increase in the baseline PHQ-9 score, women sent 29.5 fewer messages. This is a small effect in absolute terms; however, it speaks to the potential need for more personalized interventions to maximize user engagement. Most studies on digital mental health apps for common mental disorders such as depression do not report detailed use and usability metrics [37]; however, there is some evidence that also suggests a negative relationship between depression severity and engagement [38].

Users pointed to several positive features of Zuri, including feeling connected to someone who cares while having the benefit of perceived anonymity and privacy of chatting with a machine. This is consistent with existing research showing that people may be more willing to disclose personal information when they believe their responses are not being observed by another person [39], and it probably helps to explain our recruitment experience. More than a quarter of the women who completed the automated screening endorsed having recent suicidal ideation, nearly all of whom accepted our referral to in-person services. Despite having recent and regular contact with antenatal or postpartum medical providers, these women were reporting something to Zuri that they presumably had not reported to frontline medical workers—either because they were not asked, chose not to disclose, or both. There is a substantial latent need for mental health treatment that exists alongside the manifest gaps in access that chatbots such as Zuri could discover and begin to address.

In addition to reporting largely positive impressions of Zuri, users reported modest improvements in mood. To estimate this improvement, we used a multiple baseline design with repeated measures data collection and fit a multilevel model. Importantly, for making a causal inference, we did not observe an increasing trend in mood during the baseline period. We did, however, observe a small effect in the treatment period. With 432 mood ratings from 12 women before and after beginning the treatment, we estimated that mood improved by 7.0% over the average mood reported at the start of the baseline period (*d*=0.17). We have high confidence that this effect is greater than zero; however, we are similarly confident that the effect is small. We cannot conclude with confidence that this effect is indeed causal; however, quantifying this effect estimate gives us a benchmark for assessing progress in future iterations of the service. We will look to replicate and hopefully increase this effect in an RCT with a clinically indicated group of users.

We can also look to the digital health and psychotherapy literature for external benchmarks. Although there has been a proliferation of conversational agents for health in recent years [40], the evidence base is small [41]. Two recent RCTs of CBT-based chatbots stand out. In a study of 75 US college students, Tess, an automated chatbot that provides brief psychological interventions over common communication channels such as SMS and Facebook Messenger, reduced the depression symptom severity by roughly 20%, with a reported standardized effect of 0.68 [42]. Another chatbot called Woebot, a stand-alone app that delivers CBT, was tested in a trial with 70 students in the United States. Woebot reduced symptoms of depression by 19%, with a reported standardized effect of 0.44 [43]. For reference, a recent meta-analysis reported that standardized effects of traditional in-person psychotherapy for depression range from 0.66 to 0.77 [44]. Automated conversational agents such as Zuri, Tess, and Woebot have the potential to lower the cost of service delivery while expanding our reach, which could make them highly cost-effective.

Before testing this hypothesis with Zuri, however, we need to build a more robust intervention. As expected with an alpha version, we observed many opportunities for improvement. Some challenges users reported, such as the use of 2 short codes and a confusing registration process, were unique to the setup of this particular study and will not be used again. The bigger challenge will be to make the content more engaging to reduce churn and make the service more robust to misunderstandings. One way to avoid some of the confusion we observed in conversations is to move away from SMS, which can jumble the message order, and instead add a new channel through WhatsApp, the most popular messaging app in Africa [45]. In the short term, this might limit access owing to the cost of internet connectivity; however, penetration rates continue to climb rapidly. From September 2018 to September 2019, the number of data subscriptions in Kenya increased by 23% from 42.2 million to 52 million [46].

In terms of study procedures, we observed a response rate of 13.3% (86/647) among a group of women already enrolled in their county’s health SMS program. Seventeen percent of women who completed the screening scored at or above the cutoff for possible depression, and 79% (41/52) of eligible women completed the enrollment process. Depression was not a requirement for inclusion in this study; however, it will be in future studies. Our experience in this prepilot study suggests an overall enrollment rate of 1%, taking depression symptoms into account. Therefore, to recruit a sample of 100 possibly depressed pregnant women and new mothers in a future trial using the same remote procedures, these estimates suggest that we would need to advertise to a pool of at least 10,000 women. This can be easily achieved through print and digital advertising. In Nairobi County alone, there were more than 130,000 live births in 2017 [47].

Our experience with remote automated data collection suggests that women were willing and able to reply to a 1-question prompt asking them to rate their current mood. However, we were less successful at obtaining end-line data using the PHQ-9. In a future trial, it will be important to budget and plan for study staff to augment remote data collection procedures.

### Limitations

The objective of this prepilot study was to adapt *Thinking Healthy* for delivery through Zuri for developing and testing study procedures to inform the design of a future trial and to generate preliminary evidence to guide the next round of Zuri’s development. We were limited in our pursuit of these objectives given that we only offered screening and conversations in English. This likely constrained our recruitment efforts as non–English-speaking women did not have the opportunity to participate. This implies that our estimates for future recruitment are conservative. The other main limitation of operating Zuri in English is that we do not have data on how Zuri functions in Swahili. This is a priority target for development. A related limitation is that, by virtue of requiring advanced language skills, we recruited a highly educated sample of women relative to the general population. In a future trial, it will be important to explore how women of all educational backgrounds engage with Zuri.

### Conclusions

We determined that Zuri is feasible to deliver via SMS and acceptable to a sample of pregnant women and new mothers recruited from 2 large public hospitals in Kenya. The results of this prepilot study will serve as a baseline for future studies in terms of recruitment, data collection, and outcomes. The next step in Zuri’s development is to refine the intervention content and add Swahili language support. Conversational agents such as Zuri have great potential to address the large treatment gap that exists in many low-resource settings, both as a new channel of treatment and as an adjunct to traditional and task-shifting approaches.

## Appendix

Multimedia Appendix 1 Example Healthy Moms session.

Multimedia Appendix 2 Journal content related to example Healthy Moms session.

## Abbreviations

AI: artificial intelligence
CBT: cognitive behavioral therapy
LMIC: low- and middle-income country
mhGAP: Mental Health Gap Action Programme
PHQ-9: Patient Health Questionnaire–9
RCT: randomized controlled trial
