# Supplementary material for: Expanding Access to Perinatal Depression Treatment in Kenya Through Automated Psychological Support: Development and Usability Study
Source: JMIR Form Res. 2020 Oct 5;4(10):e17895. doi: 10.2196/17895 (PMC7573703; doi:10.2196/17895)
Supplement: Multimedia Appendix 1 [file formative_v4i10e17895_app1.pdf]

| Step | Message | Response Type | Next Step |
|---|---|---|---|
| 1 | Hi <<USER>>. I prepared Session 3 for you. Are you ready to start? | Closed | If YES, 4<br>If NO, 2 |
| 2 | OK, no problem. I will be ready when you are ready. Just text me START to get started. | None | 3 |
| 3 | You can also text me a question or say HeyZuri if you just want to chat. I'm here for you. | None | END |
| 4 | Let's start by reviewing what we talked about in the last session. | None | 5 |
| **Task 1: Review previous session** | | | |
| 5 | What is one thing you learned from our last session? | Open | 6 |
| 6 | In the last session we worked on thinking positively about your relationship with your baby. | None | 7 |
| 7 | I understand that motherhood can seem challenging. It can be difficult to always think positively about your baby in your womb. | None | 8 |
| 8 | Have you had any unhealthy thoughts about your relationship with your baby since we last spoke? | Closed | If YES, 9<br>If NO, 10 |
| 9 | That's okay. None of these thoughts make you a bad mother. | None | 11 |
| 10 | Good to hear! If you find yourself having unhealthy thoughts, remember that these do not make you a bad mother. | None | 11 |
| 11 | We practiced how to change these unhealthy thoughts to healthier ones. | None | 12 |
| 12 | Do you remember some positive ways to think about your relationship with your baby? | Closed | 13 |
| 13 | Positive thoughts reduce guilt, helping both you and your baby to be healthier. | None | 14 |
| **Task 2: Review mood ratings** | | | |
| 14 | Before we begin, would you say you've had more good days or bad days since our last session? | Closed | If POS, 15<br>If NEG, 18 |
| 15 | I'm glad you have been having more good days! | None | 16 |
| 16 | What is one thing that made you happy this week? | Open | 17 |
| 17 | Nice. | None | 21 |
| 18 | I'm sorry. What is something that made you have a bad day? | Open | 19 |
| 19 | Thank you for sharing. | None | 20 |
| 20 | It can be hard to always stay positive, but hopefully today we'll continue learning to think healthier thoughts. | None | 23 |
| 21 | For the next part of our session, we'll learn to recognize negative thoughts and change them to positive ones. | None | 22 |
| 22 | This is a great skill to have, even when we have been having good days. | None | 23 |
| 23 | Are we together? | Closed | If YES, 24<br>If NO, END |
| 24 | Great. Let us continue! | None | 25 |
| **Task 3: Teach new skills** | | | |
| 25 | Why is it important to have positive relationships with the people close to you? | Open | 26 |
| 26 | The people we surround ourselves with can support us during pregnancy and childbirth. | None | 27 |
| 27 | But, it can be difficult to maintain positive relationships when you're stressed. | None | 28 |

| Step | Message | Response Type | Next Step |
|---|---|---|---|
| 28 | Do you ever feel isolated from others? | Closed | 29 |
| 29 | When we feel isolated from others, we start to avoid people. This creates more distance between us and our family and friends, making us feel even more isolated. | None | 20 |
| 30 | Have you ever felt like there's no use meeting new people? | Closed | If YES, 31<br>If NO, 32 |
| 31 | Such feelings are normal and can develop when we experience stress and problems. | None | 32 |
| 32 | If you feel this way later, know that such feelings are normal and can develop when we experience stress and problems. | None | 33 |
| 33 | If we do nothing, we will feel even worse. | None | 34 |
| 34 | Replacing unhealthy thinking with helpful thinking can pull us out of this mood. Let's practice how! | None | 35 |
| 35 | Are you excited, or are you really excited? | Closed | 36 |
| 36 | Today we'll practice ways to make thinking healthy about your relationships a little easier. | None | 37 |
| 37 | What is something you can say to yourself if you're feeling like there's no use meeting people? | Open | 38 |
| 38 | Big interactions can be intimidating, but maybe you can focus on talking to a few people you like! | None | 39 |
| 39 | If you talk to even one or two people, you can share your feeling and maintain social relationships. | None | 40 |
| 40 | Another common unhealthy thought is that "an expecting mother should not be going out of the house". How can we change this to a positive thought? | Open | If NEG, 41<br>If POS, 42 |
| 41 | As long as you are in a safe and healthy environment, you should continue to interact with others outside the home. | None | 43 |
| 42 | Good idea. As long as you are in a safe and healthy environment, you should continue to interact with others outside the home. | None | 43 |
| 43 | It's important to remember that you are not alone. | None | 44 |
| 44 | Family members should also recognize that social support helps the baby develop. | None | 45 |
| 45 | I would like to review a few activities to practice thinking and acting healthy. Are you ready to practice? | Closed | If YES, 46<br>If NO, 47 |
| 46 | OK, great. | None | 48 |
| 47 | OK, just tell me when you're ready to begin again. | None | 48 |
| 48 | Who are the main people within your family who can provide you with support? | Open | If POS, 49<br>If NEG, 50 |
| 49 | That's wonderful! Family can help us even during the hardest times. | None | |
| 50 | Do you have friends in the community who can support you during your pregnancy? | Closed | If YES, 51<br>If NO, 52 |
| 51 | That's great! Many times, our friends share similar experiences to us. | None | 52 |
| 52 | We don't need a lot of people. Even just one good friend can make a tough time easier. | None | 53 |
| 53 | Tell me about a time when a friend helped you through a tough situation. | Open | 54 |
| 54 | Positive relationships with your family and community members will provide support throughout your pregnancy. | None | 55 |

| Step | Message | Response Type | Next Step |
|---|---|---|---|
| 55 | Now, let us go over the other activities you have been working on. | None | 56 |
| 56 | Were you able to make any recordings in your diet chart since our last session? | Closed | If YES, 57<br>If NO, 58 |
| 57 | Wonderful!! Keep it up. | None | 59 |
| 58 | It can be hard to find time in the day, but keep trying because self-care is so important. | None | 59 |
| 59 | Eating a good, balanced diet is one of the best things you can do for yourself and your baby. | None | 60 |
| 60 | What did you enjoy most with your diet this week? | Open | 61 |
| 61 | Finding ways to have fun with your diet will help keep you motivated! | None | 62 |
| 62 | Keep up the good work! | None | 63 |
| 63 | Were you able to make any recordings in your rest and relaxation chart since our last session? | Closed | If YES, 64<br>If NO, 65 |
| 64 | Way to go! | None | 66 |
| 65 | Tracking is a really helpful tool when we are learning to try new things. Keeping a record helps us to see progress. | None | 66 |
| 66 | Have you been able to practice any of the relaxation exercises since our last session? | Closed | If YES, 67<br>If NO, 68 |
| 67 | You're doing very well! | None | 69 |
| 68 | The good news is that you only need to find a few minutes a day to start improving your health and mood. | None | 69 |
| 69 | You can practice your way to good health habits that will make you and your baby feel great! | None | 70 |
| **Task 4: Introduce practice-based homework** | | | |
| 70 | Are you starting to feel more comfortable with the homework? | Closed | If YES, 71<br>If NO, 72 |
| 71 | That's what I like to hear! | None | 73 |
| 72 | Over time, this will start to get easier. | None | 73 |
| 73 | Continue to rate your mood in your calendar. Additionally, continue to fill out your diet chart. | None | 74 |
| 74 | Make sure you're also following the rest and relaxation activities! | None | 75 |
| 75 | How does this plan sound to you so far? | Open | 76 |
| 76 | This time, I want you to try a new activity to help with strengthing social relationships. | None | 77 |
| 77 | Is it possible for you to form a group with other pregnant mothers in your area? This can be in person or online! | Closed | If YES, 78<br>If NO, 79 |
| 78 | Wonderful! You could call this group the Mother and Baby Health group, and you could talk once a week about anything! | None | 80 |
| 79 | That's okay, it's a pretty big task. Instead of a big group, try finding one other woman to share your experiences with, and hear her experieces as well! | None | 80 |
| 80 | Our next session will begin in 1 week. I'll remind you when it's time to start. If you want to chat before then, just text HeyZuri to 40225. | None | END |
